# Clocks slide rather than freeze during torpor in the mouse

**DOI:** 10.1016/j.isci.2026.116402

**Published:** 2026-06-17

**Authors:** Timna Hitrec, Ludovico Taddei, Lukasz Chrobok, Megan Elley, William S.R. Wheatley, Anthony E. Pickering, Michael T. Ambler

**Affiliations:** 1 School of Physiology, Pharmacology and Neuroscience, University of Bristol, Bristol, UK; 2 Department of Biomedical and Neuromotor Sciences, University of Bologna, Bologna, Italy

**Keywords:** Molecular mechanism of behavior, Behavioral neuroscience, Systems neuroscience

## Abstract

Torpor is an energy-saving strategy used by animals in response to environmental challenges. Torpor is subject to circadian influence, but the effect of torpor itself on circadian rhythms is poorly understood. We investigated whether torpor alters circadian behavior by assessing locomotor activity after torpor in mice emerging into constant darkness (without Zeitgebers). Torpor was induced by food deprivation or by chemogenetic re-activation of “torpor-active” preoptic area neurons. In both conditions, torpor did not disrupt circadian rhythmicity, although a significant phase advance of ∼40 min was associated with arousal from fasting-induced torpor. Increased wheel-running activity occurred before torpor, likely reflecting food-seeking behavior. After emergence from fasting-induced torpor, locomotor activity decreased, while food/water intake increased to balance any nutritional deficit back to pre-torpor levels. Together, these findings suggest that torpor, and the associated hypothermia, does not disturb circadian function and that torpor (or arousal) acts as a non-photic phase advance stimulus.

## Highlights

• Circadian rhythms are preserved during torpor in mice
• Fasting-induced torpor acts as a non-photic cue that advances circadian activity phase
• Synthetic torpor does not alter circadian timing
• Arousal from torpor acutely increases feeding and reduces locomotor activity

## Graphical abstract

**Figure:**
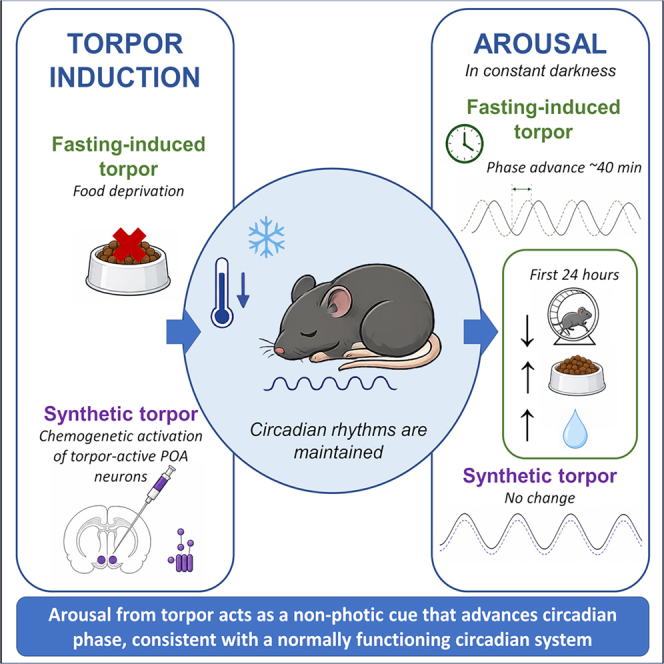

## Introduction

Torpor and hibernation are energy-conserving physiological states that allow animals to survive periods of food scarcity or challenging environmental conditions by transiently reducing metabolic rate, body temperature, and activity.^1^ These states interact with systems that regulate energy homeostasis, including circadian rhythmicity. Circadian clocks coordinate daily patterns of activity, feeding, and metabolism in relation to the light-dark cycle and are known to influence the timing of torpor. In small rodents, torpor entry and arousal are not randomly distributed across the day. Torpor entry tends to occur toward the end of the dark (active) phase and with a high degree of reproducibility across successive days,^2^^,^^3^^,^^4^^,^^5^^,^^6^ whereas arousal tends to occur in the light (inactive) phase. However, how engagement of torpor feeds back onto circadian timing remains poorly understood. Importantly, despite mice being the primary model organism in circadian research, there is currently limited evidence on how daily torpor affects circadian timing in this species. A key feature of the circadian clock is temperature compensation—the ability to maintain a relatively stable period across a range of temperatures despite the strong temperature dependence of most biochemical reactions.^7^^,^^8^^,^^9^^,^^10^ This property implies that circadian timing mechanisms may be inherently resistant to moderate changes in body temperature. In seasonal hibernators, daily oscillations of activity and body temperature are markedly attenuated or even absent during prolonged torpor bouts, re-emerging only upon arousal.^11^^,^^12^ Such findings have been interpreted as evidence that profound and sustained hibernation can suppress circadian outputs; however, to our knowledge, there is no evidence on what happens to circadian clock function in daily torpor. Moreover, while temperature compensation of the circadian clock is well established *in vitro*, it remains unclear whether this property holds *in vivo* under conditions of marked hypothermia and metabolic suppression, where many physiological processes are expected to slow down. Interpreting the effects of torpor on circadian rhythms is further complicated by the behavioral and metabolic changes that accompany torpor. Fasting-induced torpor is preceded by a pronounced increase in locomotor activity, which represents a potent non-photic modulator of circadian timing.^13^^,^^14^ In addition, external zeitgebers such as light can strongly influence circadian rhythmicity following torpor or hibernation. For example, in Arctic ground squirrels, circadian rhythms suppressed during torpor rapidly reappear after arousal, and even brief light exposure shortly thereafter enhances the robustness of these resuming rhythms.^12^ Together, these factors highlight the need to disentangle the intrinsic effects of hypothermia from those driven by behavioral or environmental cues. In recent years, synthetic torpor—an artificially induced hypothermic state that reproduces key features of fasting-induced torpor—has emerged as a useful tool to address these questions. By enabling hypothermia to be induced through chemogenetic manipulation,^15^^,^^16^^,^^17^ this approach offers the potential to examine the effect of hypothermia without the confounding effect of fasting, including its impact on circadian rhythms.

In this study, we examined how both fasting-induced and synthetic torpor affect circadian rhythms in mice. Food was removed at the onset of the dark phase, shortly before mice would consume the largest meal of the day, to minimize the duration of deprivation required to induce torpor while remaining consistent with established experimental paradigm.^18^^,^^19^ Importantly, this timing also ensures that the resulting torpor bouts align with their natural circadian occurrence.^3^^,^^6^ Wheel-running activity was analyzed under constant darkness before and after torpor to determine whether torpor alters the free-running circadian period, produces shifts in circadian phase, or disrupts behavioral rhythmicity in the absence of external cues. In parallel, food and water intake and body temperature were monitored. Together, these analyses provide insight into how daily torpor interacts with circadian timing and help clarify the extent to which endogenous circadian rhythms remain resilient during hypothermic states in mice.

## Results

### Fasting-induced torpor does not disrupt intrinsic circadian patterns of activity, but advances the clock

**Figure 1:**
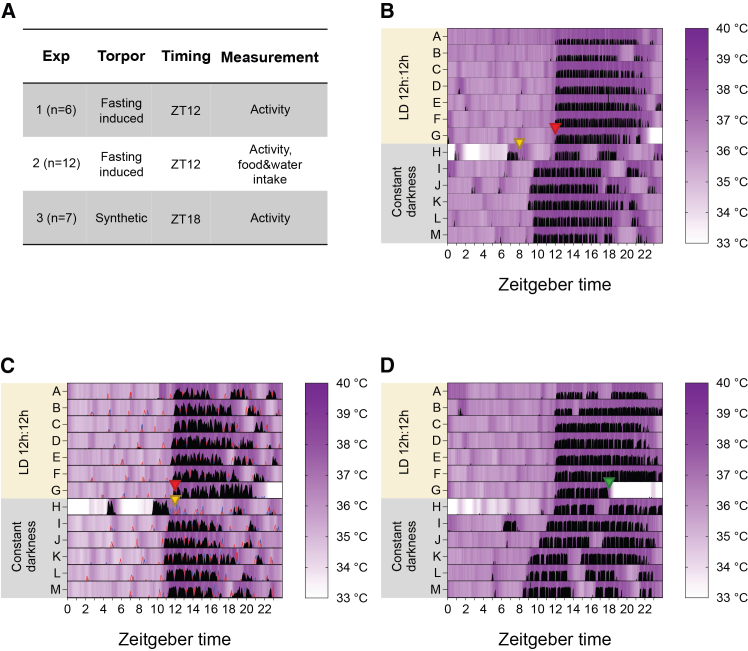
Influence of torpor on circadian behaviors (A) Summary table of the experimental conditions. (B) Representative actogram of an animal from experiment 1. Each horizontal band represents a day; core body temperature is depicted using a purple-to-white gradient, while the black trace indicates locomotor activity (also for C and D). Food was removed at ZT12 of day G (red arrow), and it entered torpor toward the end of the dark phase, indicated by the lowering of body temperature (marked in white). This mouse aroused from torpor approximately at CT6 and then engaged in vigorous locomotory activity before the food was given back (yellow arrow). (C) Representative actogram from experiment 2. Red and blue lines indicate food and water consumption, respectively. Food was removed at ZT12 of day G (red arrow). This animal expressed two torpor bouts (marked in white), engaging in vigorous locomotory activity immediately after arousing from both. Food was returned at CT12 (yellow arrow). (D) Representative actogram from a Torpor-TRAP mouse in experiment 3. Clozapine N-oxide hydrochloride (CNO) was injected to induce synthetic torpor at ZT18 of day G (green arrow). This animal entered torpor shortly after injection and emerged in the second half of the following inactive phase, neither engaging in vigorous locomotor activity upon arousal from torpor nor having a subsequent period of inactivity over the following 24 h.

**Figure 2:**
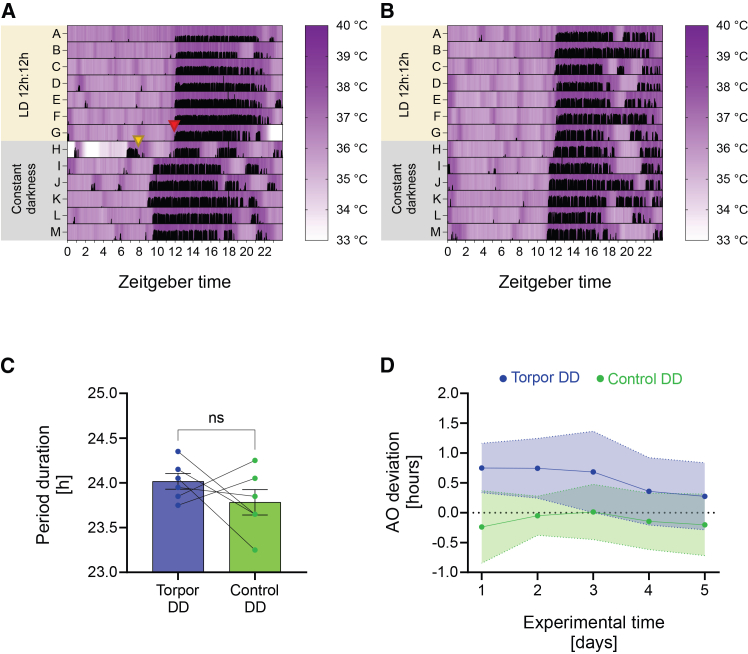
Experiment 1 circadian rhythmicity (A) Representative actogram for the Torpor DD condition. Each horizontal band represents a day. Food was removed at ZT12 of day G (red arrow) and returned at CT8 the following day (yellow arrow). Core body temperature is depicted using a purple-to-white gradient, while the black trace indicates locomotor activity. For the first 7 days (A–G), the animal was exposed to a 12 h:12 h LD cycle. Starting from day H, the animal was maintained in constant darkness for the following week. (B) Representative actogram for the Control DD condition for the same animal represented in (A). *Ad libitum* access to food was maintained, but conditions were otherwise identical. (C) Free-running period of locomotor activity in Torpor DD and Control DD conditions (*n* = 6). Data are presented as mean ± SEM; dots represent individual values. Data were compared through a paired *t* test. Torpor DD: 24.02 ± 0.09 h vs. Control DD: 23.78 ± 0.14 h; paired *t* test: *t*_(5)_ = 1.115, *p* = 0.316. (D) Activity-onset deviation from predicted values in the first 5 days of constant darkness in the Torpor DD condition and in the Control DD condition. Blue and green dots represent daily mean values; colored areas represent the standard error of the mean. Data were analyzed using a mixed-effect model using Time and Experimental Condition as factors. Sidak’s multiple comparisons test was then used to compare onset deviation between the two experimental conditions for each day. Statistical analysis showed no significant differences. Individual datapoints are represented in Figure S3A.

To determine whether fasting-induced torpor affects the period and phase of the endogenous circadian clock, in experiment 1, we monitored wheel-running activity in mice before torpor (under a standard 12 h:12 h light-dark cycle [LD]) and after torpor, when animals emerged in constant darkness (Torpor DD) without external zeitgebers (Figures 1 and 2A). As a control, utilizing a crossover design, wheel-running activity was also recorded in the same animals entering constant darkness fed *ad libitum* (Control DD, Figure 2B). Food withdrawal at Zeitgeber Time (ZT) 12 typically triggered torpor entry ∼11 h later (∼ZT23, Figure 2A), characterized by a pronounced drop in core body temperature and activity. Animals emerged from torpor around Circadian Time (CT) 4 (∼5 h later) and engaged in vigorous wheel running until food was returned at CT8 (yellow arrow, Figure 2A). This was followed by ∼24 h of reduced activity with markedly reduced wheel running levels despite normalized core temperature.

Comparing the free-running period (τ) in constant darkness after torpor (Torpor DD) vs. undisturbed constant darkness (Control DD) revealed no statistically significant differences (Torpor DD: 24.02 ± 0.09 h vs. Control DD: 23.78 ± 0.14 h, mean ± SEM; paired *t* test: *t*_(5)_ = 1.115, *p* = 0.316, Figure 2C). Furthermore, mixed-effect model analysis of activity onset deviation (Figure 2D; Figure S3A) showed no significant effects for the factors “Time” (F_(4,20)_ = 0.634, *p* = 0.644) or “Experimental Condition” (F_(1,5)_ = 1.149, *p* = 0.333), or for their interaction (F_(4,19)_ = 1.305, *p* = 0.304). No post hoc pairwise comparison reached statistical significance. Despite the lack of statistical significance, the difference between mean value from the two conditions was 0.67 h (∼40 min), with Torpor DD values consistently indicating a phase advance compared to Control DD. The effect size was medium for the factor Time (Cohen’s *f* = 0.36) and large for the factor Experimental Condition (Cohen’s *f* = 0.48) and their interaction (Cohen’s *f* = 0.52, Table S1). Notably, we observed a burst of wheel running activity around CT8 on the day following torpor, most likely linked to food-seeking behavior.

To minimize the potential confounding effect of food return on circadian timing observed in experiment 1, a second experimental protocol (experiment 2) involved returning food at CT12 after torpor (24 h after removal). While this timing does not eliminate the presence of a zeitgeber, it aligns it with the expected onset of the active phase and is less likely to induce a phase shift. Apart from the timing of food return, the experimental design mirrored experiment 1.

**Figure 3:**
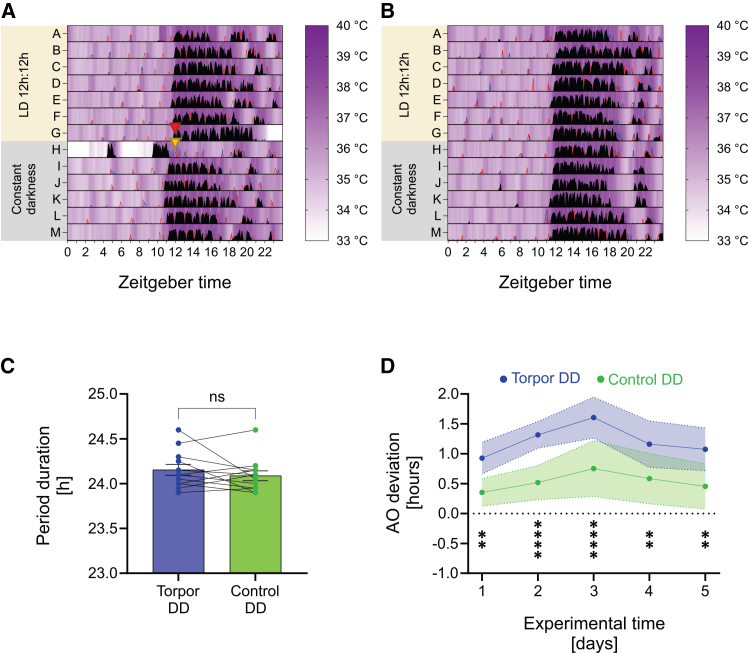
Experiment 2 circadian rhythmicity (A) Representative actogram for the Torpor DD condition. Each horizontal band represents a day. Food was removed at ZT12 of day G (red arrow) and returned at CT12 the following day (yellow arrow). Core body temperature is depicted using a purple-to-white gradient, while the black trace indicates locomotor activity. Red and blue lines indicate food and water consumption, respectively. For the first 7 days (A–G), the mouse was exposed to a 12 h:12 h LD cycle. Starting from day H, the mouse was maintained in constant darkness for the following week. (B) Representative actogram for the Control DD condition for the same animal represented in (A). *Ad libitum* access to food was maintained, but experimental conditions were otherwise identical. (C) Free-running period of locomotor activity in Torpor DD and Control DD conditions (*n* = 12). Data are presented as mean ± SEM; dots represent individual values. Data were compared through a paired *t* test. Torpor DD: 24.15 ± 0.06 h vs. Control DD: 24.1 ± 0.06 h; paired *t* test: *t*_(11)_ = 1.076, *p* = 0.305. (D) Activity-onset deviation from predicted values in the first 5 days of constant darkness in the Torpor DD condition and in the Control DD condition. Blue and green dots represent daily mean values; colored areas represent the standard error of the mean. Data were analyzed using a 2-way repeated measures ANOVA, using Time and Experimental Condition as factors. Sidak’s multiple comparisons test was then used to compare onset deviation between the two experimental conditions for each day. ∗∗∗∗ = *p* < 0.0001, ∗∗ = *p* < 0.05 in post hoc pairwise comparisons. Individual datapoints are represented in Figure S3B.

Consistent with experiment 1, food withdrawal at ZT12 triggered torpor entry ∼11 h later (∼ZT23, Figure 3A). However, emergence from torpor was less synchronized than in the previous experiment; animals displayed a variable number of torpor bouts of differing durations. Crucially, emergence from the final torpor bout consistently occurred during the second half of the subjective day (when lights would normally be on), between CT7 and CT11. Following emergence, animals engaged in vigorous wheel running until food was returned at CT12. Similar to experiment 1, no significant difference in wheel-running period was found between the two DD conditions (Torpor DD: 24.15 ± 0.06 h vs. Control DD: 24.1 ± 0.06 h, mean ± SEM; paired *t* test: t_(11)_ = 1.076, *p* = 0.305, Figure 3C). However, the two-way repeated measures ANOVA for activity onset deviation (Figure 3D; Figure S3B) revealed significant main effects for both Time (F_(4,44)_ = 3.429, *p* = 0.016) and Experimental Condition (F_(1,11)_ = 27.04, *p* < 0.001), and the interaction was not significant (F_(4,44)_ = 0.631, *p* = 0.643). The effect size was large for both the Time (Cohen’s *f* = 0.56) and Experimental Condition (Cohen’s *f* = 1.57) factors, while it was small for their interaction (Cohen’s *f* = 0.24, Table S1). All post hoc pairwise comparisons between the two conditions were statistically significant. The difference between mean values from the two conditions was 0.68 h (∼41 min), with Torpor DD values consistently indicating a phase advance compared to Control DD. These results indicate that torpor induces a significant phase advance in wheel-running behavior without altering its period.

Experiment 2 allowed us to mitigate the confounding influence of food return timing, leaving other potential entraining stimuli, such as the torpor bout itself or the subsequent hyperactivity bout, intact. Consequently, for both experiments, we analyzed potential correlations between the magnitude of the phase shift (regardless of statistical significance) and the following parameters describing torpor: torpor latency (time from food deprivation to torpor onset), torpor index (indicator of torpor depth and duration), and the timing of the post-torpor hyperactivity bout (relative to the last bout in animals that showed multiple torpor bouts). The phase-shift value for each animal was calculated by averaging the deviation of predicted vs. actual activity onset over the 5 days post-torpor. No significant correlations were identified.

### Fasting-induced torpor causes an acute increase in locomotor activity

**Figure 4:**
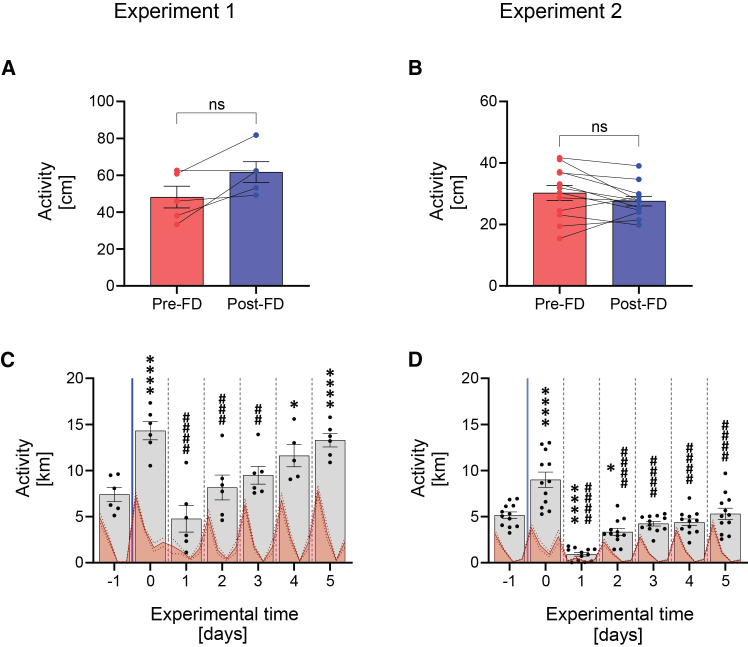
Locomotor activity in experiments 1 and experiment 2 (A) Cumulative locomotor activity in the 6 days preceding and following the time of food deprivation in experiment 1 (*n* = 5). Data are presented as mean ± SEM; dots represent individual values. Data were compared through a paired *t* test. Pre-FD: 48.16 ± 5.89 km vs. post-FD: 61.72 ± 5.63 km; paired *t*_(4)_ = 2.497, *p* = 0.067. (B) Similar plot of cumulative locomotor activity in the 6 days preceding and following the time of food deprivation in experiment 2 (*n* = 12). Data are presented as mean ± SEM; dots represent individual values. Data were compared through a paired *t* test. Pre-FD: 31.44 ± 2.70 km vs. post-FD: 28.38 ± 2.01 km; paired *t*_(11)_ = 2.088, *p* = 0.057. (C) Locomotor activity in the day preceding and across the 6 days following the time of food deprivation (measured from CT12) in experiment 1. Gray histogram bars represent locomotor activity as daily bins; red line/area represents locomotor activity as 6-h bins, and black dots represent individual daily values. Baseline values (day −1) were derived from the average of the 6 days preceding treatment. Blue solid vertical line represents the moment of food deprivation (ZT12). Black dotted lines represent each day CT12. Data are shown as mean ± SEM. Data were analyzed using a mixed-effect model for repeated measures. Post hoc comparisons were performed using Sidak’s test. The following comparisons were made: baseline (day −1) was compared with torpor day (day 0) and with each of the subsequent five days (days 1–5) (∗∗∗∗ = *p* < 0.0001, ∗ = *p* < 0.05); additionally, torpor day (day 0) was compared with each of the subsequent 5 days (days 1–5) (#### = *p* < 0.0001, ### = *p* < 0.001, ## = *p* < 0.01). (D) Locomotor activity in the day preceding and in the 6 days following the time of food deprivation (measured from ZT12) in experiment 2. Plotted as (C). Data are shown as mean ± SEM. Data were analyzed using a one-way repeated measures ANOVA. Post hoc comparisons were performed using Sidak’s test. The following comparisons were made: baseline (day −1) was compared with torpor day (day 0) and with each of the subsequent five days (days 1–5) (∗∗∗∗ = *p* < 0.0001, ∗ = *p* < 0.05); additionally, torpor day (day 0) was compared with each of the subsequent 5 days (days 1–5) (#### = *p* < 0.0001).

We next assessed locomotor activity to determine whether torpor altered overall or daily wheel-running behavior (Figure 4). Cumulative activity, summed across the 6 days before and 6 days after the day of fasting-induced torpor (including torpor day in the 6 days comprising the “post-Food Deprivation (FD)” period), did not differ significantly in either experiment 1 (pre-FD: 48.16 ± 5.89 km vs. post-FD: 61.72 ± 5.63 km, mean ± SEM; paired *t*_*(*4)_ = 2.497, *p* = 0.067, Figure 4A) or experiment 2 (pre-FD: 31.44 ± 2.70 km vs. post-FD: 28.38 ± 2.01 km, mean ± SEM; paired *t*_(11)_ = 2.088, *p* = 0.057, Figure 4B). It is worth noting that, although both comparisons fail to reach statistical significance by a narrow margin, the effect of fasting-induced torpor is opposite in the two experiments, supporting the conclusion that there is no biologically meaningful difference in cumulative locomotor activity.

Analysis of daily activity revealed marked changes surrounding the torpor episode. On the day of torpor (day 0), mice displayed a significant increase in wheel running after food withdrawal but before torpor onset, nearly doubling baseline levels in both experiment 1 (day −1: 7.41 ± 0.78 km vs. day 0: 14.33 ± 0.98 km, mean ± SEM; Sidak’s post hoc, *p* < 0.001; Figure 4C) and experiment 2 (day −1: 5.24 ± 0.45 km vs. day 0: 9.68 ± 0.88 km, mean ± SEM; Sidak’s post hoc, *p* < 0.001; Figure 4D). In contrast, activity decreased greatly on the day following torpor and then gradually returned to baseline levels over the subsequent days in both experiments (Figures 3C and 3D).

Under baseline conditions, activity was concentrated within the active phase, as expected. During the torpor day, however, activity was markedly increased during the rest phase, due to the locomotor hyperactivity bout occurring immediately after arousal (Figures 4C and 4D). No major changes in temporal distribution were detected in the days thereafter.

### Compensatory changes in post-torpor feeding and drinking

**Figure 5:**
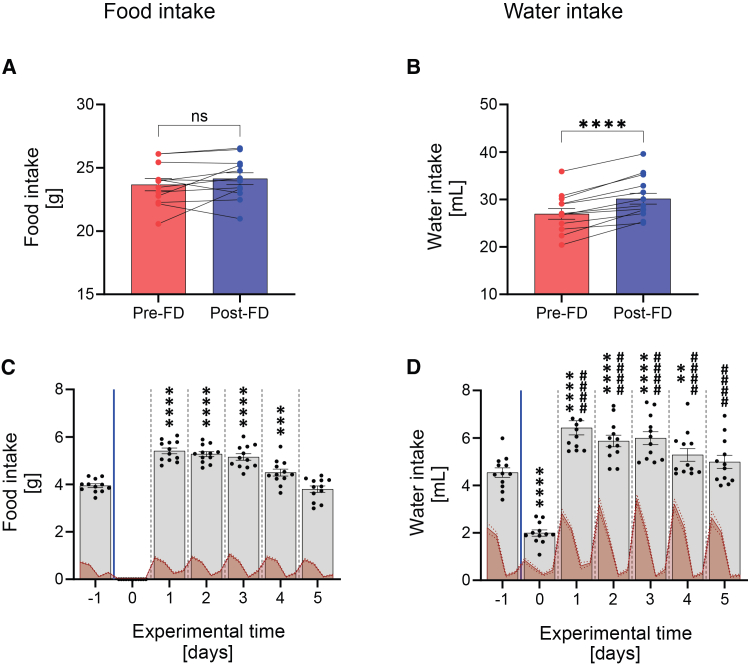
Food and water intake in experiment 2 (A) Cumulative food intake in the 6 days preceding and following the time of food deprivation in experiment 2 (*n* = 12). Data are presented as mean ± SEM; dots represent individual values. Data were compared through a paired *t* test. Pre-FD: 23.57 ± 0.48 g vs. post-FD: 23.99 ± 0.43 g, mean ± SEM; paired *t*_(11)_ = 0.941, *p* = 0.364. (B) Equivalent plot of cumulative water intake in the 6 days preceding and following the time of food deprivation in experiment 2 (*n* = 12). Data were compared through a paired *t* test. Pre-FD: 26.95 ± 1.11 mL vs. post-FD: 30.14 ± 1.13 mL, mean ± SEM; paired *t*_(11)_ = 6.467, ∗∗∗∗ = *p* < 0.001. (C) Food intake in the day preceding and in the 6 days following the time of food deprivation (measured from ZT12) in experiment 2 (*n* = 12). Gray histograms represent food intake as daily bins; red line/area tracks food intake as 6-h bins; black dots represent individual daily values. Baseline values (day −1) were derived from the average of the 6 days preceding treatment. Blue solid vertical line represents the moment of food deprivation (ZT12). Black dotted lines represent each day CT12. Data are shown as mean ± SEM. Data were analyzed using a repeated measures one-way ANOVA. Post hoc comparisons were performed using Sidak’s test. Baseline (day −1) was compared with each of the 5 days following torpor day (days 1–5). ∗∗∗∗ = *p* < 0.0001, ∗∗∗ = *p* < 0.001. (D) Equivalent plot of water intake in the day preceding and in the 6 days following the time of food deprivation (measured from ZT12) in experiment 2. Data are shown as mean ± SEM. Data were analyzed using a repeated measures one-way ANOVA. Post hoc comparisons were performed using Sidak’s test. Baseline (day −1) was compared with torpor day (day 0) and with each of the subsequent 5 days (days 1–5) (∗∗∗∗ = *p* < 0.0001, ∗∗ = *p* < 0.01); additionally, torpor day (day 0) was compared with each of the subsequent 5 days (days 1–5) (#### = *p* < 0.0001).

Food and water intake were monitored before, during, and after the period of torpor (Figure 5). Cumulative food consumption summed over the 6 days preceding and following the day of food deprivation (including the torpor day, where no food was available) did not differ significantly (pre-FD: 23.57 ± 0.48 g vs. pFD: 23.99 ± 0.43 g, mean ± SEM; paired *t*_(11)_ = 0.941, *p* = 0.364, Figure 5A), indicating stable intake across the period before and after food deprivation. Notably, cumulative water intake was significantly elevated during the recovery period compared with baseline (pre-FD: 26.95 ± 1.11 mL vs. post-FD: 30.14 ± 1.13 mL, mean ± SEM; paired *t*_(11)_ = 6.467, *p* < 0.001, Figure 5B).

A finer-grain, day-by-day, analysis showed that during the day of torpor, water intake was markedly reduced relative to baseline (paralleling the mandatory period of food withdrawal, Figures 4C and 4D). In the subsequent 4 days, both food and water intake were significantly increased, consistent with a compensatory rebound (Figures 4C and 4D). The largest magnitude effect was seen on day 1 for food intake (day −1: 3.93 ± 0.08 g vs. day 1: 5.42 ± 0.11 g, mean ± SEM; Sidak’s post hoc, *p* < 0.001), and similarly for water intake (day −1: 4.49 ± 0.19 mL vs. day 1: 6.40 ± 0.28 mL, mean ± SEM; Sidak’s post hoc, *p* < 0.001).

Feeding and drinking behavior largely maintained their temporal distribution, with consumption concentrated in the active phase (Figures 4C and 4D). The only exception was a modest increase in both food and water intake during the rest phase on the day immediately following torpor.

### Synthetic torpor does not affect circadian rhythms

**Figure 6:**
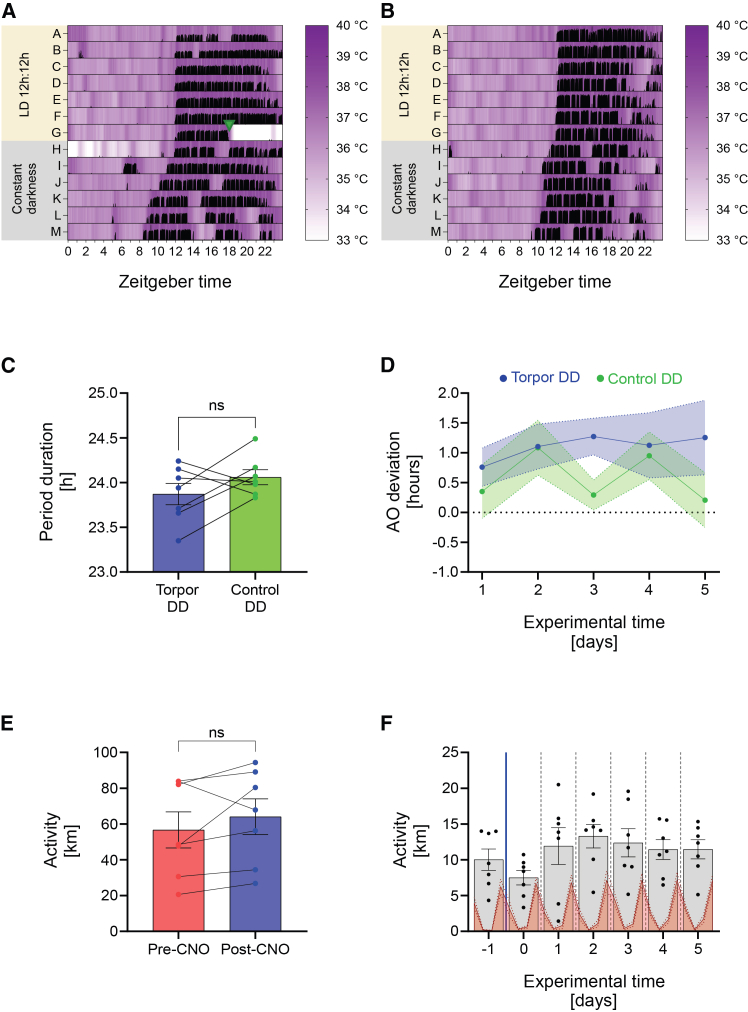
Experiment 3 results (A) Representative actogram for the Torpor DD condition. Each horizontal band represents a day. CNO was administered at ZT18 of day G (green arrow). *Ad libitum* access to food was maintained. Core body temperature is depicted using a purple-to-white gradient, while the black trace indicates locomotor activity. For the first 7 days (A–G), the mouse was exposed to a 12 h:12 h LD cycle. Starting from day H, the mouse was maintained in constant darkness for the following week. (B) Equivalent representative actogram for the Control DD condition (no CNO injection) for the same mouse represented in (A). (C) Free-running period of locomotor activity in Torpor DD and Control DD conditions (*n* = 7). Data are presented as mean ± SEM; dots represent individual values. Data were compared through a paired *t* test. Torpor DD: 23.87 ± 0.12 h vs. Control DD: 24.06 ± 0.22 h; paired *t* test: *t*_(6)_ = 1.357, *p* = 0.224. (D) Activity-onset deviation from predicted values in the first 5 days of constant darkness in the Torpor DD condition and in the Control DD condition (*n* = 7). Blue and green dots represent daily mean values; colored areas represent the standard error of the mean. Data were compared using a mixed-effect model for repeated measures, with Time and Experimental Condition as factors. Sidak’s multiple comparisons test was then used to compare onset deviation between the two experimental conditions for each day. Individual datapoints are represented in Figure S3C. (E) Cumulative locomotor activity in the 6 days preceding and following the time of CNO injection in experiment 3 (*n* = 7). Data are presented as mean ± SEM; dots represent individual values. Data were compared through a paired *t* test. Pre-CNO: 56.78 ± 10.07 km vs. post-CNO: 64.18 ± 5.42 km; paired *t*_(6)_ = 1.365, *p* = 0.221. (F) Locomotor activity in the day preceding and in the 6 days following the time of food deprivation (measured from ZT12) in experiment 3. Gray histograms represent locomotor activity as daily bins; red line/area represent locomotor activity as 6-h bins, and black dots represent individual daily values. Baseline values (day −1) were derived from the average of the 6 days preceding treatment. Blue solid vertical line represents the moment of CNO injection (ZT18). Black dotted lines represent each day CT12. Data are shown as mean ± SEM. Data were analyzed using a mixed-effect model for repeated measures. Post hoc comparisons were performed using Sidak’s test. The following comparisons were made: baseline (day −1) was compared with torpor day (day 0) and with each of the subsequent 5 days (days 1–5); additionally, torpor day (day 0) was compared with each of the subsequent 5 days (days 1–5). Statistical analysis showed no significant differences.

To remove the potential confounding influence of food availability and food return as zeitgebers, without any need for a period of fasting, we induced torpor in a cohort of torpor-TRAP mice using Clozapine N-oxide hydrochloride (CNO) administration at ZT18 and monitored their activity and core body temperature. CNO injection at ZT18 triggered torpor entry ∼1 later (∼ZT19, Figure 6A). In contrast to experiments 1 and 2, animals did not display hyperactivity bouts upon arousal, likely due to immediate food access.

Comparison of free-running period did not reveal statistically significant differences between the two experimental conditions (Torpor DD: 23.87 ± 0.12 h vs. Control DD: 24.06 ± 0.22 h, mean ± SEM; paired *t* test: *t*_*(*6)_ = 1.357, *p* = 0.224, Figure 6C), which was in keeping with experiments 1 and 2. Mixed-effect model analysis of activity onset deviation (Figure 4D; Figure S3C) showed no significant effect, neither for the factors Time (F_(4,24)_ = 0.641, *p* = 0.638) and Experimental Condition (F_(1,6)_ = 2.171, *p* = 0.191) nor for the interaction of the two (F_(4,22)_ = 1.160, *p* = 0.355). Effect sizes were moderate for Time (Cohen’s *f* = 0.33) and large for Experimental Condition (Cohen’s *f* = 0.60) and their interaction (Cohen’s *f* = 0.46, Table S1). No pairwise comparisons reached statistical significance. However, consistent with experiments 1 and 2, the post-torpor condition was characterized by a consistent phase advance for activity onset. The effect did not reach statistical significance, and the magnitude of the observed shift (mean difference of 0.61 h, ∼32 min) was smaller than that observed in other experiments.

Cumulative locomotor activity across the 5 days of baseline and 5 days of recovery showed no significant changes (pre-CNO: 56.78 ± 10.07 km vs. post-CNO: 64.18 ± 5.42 km, mean ± SEM; paired *t*_(6)_ = 1.365, *p* = 0.221, Figure 6E). Daily analysis (Figure 6F) revealed a modest reduction in activity during torpor and a slight increase in the following days, although these differences were not statistically significant. Overall, synthetic torpor had minimal impact on locomotor behavior.

## Discussion

Relatively little is known about how daily torpor, either natural or synthetic, influences circadian rhythms in small mammals. In hibernators, several studies have shown disruption or even transient suppression of circadian rhythmicity. For example, in European hamsters, circadian rhythmicity essentially ceases during deep hibernation, resuming only after arousal.^11^ In ground squirrels, circadian activity rhythms are dampened during torpor but recover upon arousal.^12^ However, to our knowledge, no comparable data have been obtained for mice. This is notable given that mice represent the most widely used model organism in circadian biology, yet the impact of daily torpor on circadian timing has remained largely unexplored. Furthermore, mice are frequently used to study the effects of calorie restriction, using protocols that would induce torpor (even if the investigators do not recognize this^20^). These results provide new insights, showing that both fasting-induced and synthetic torpor in mice largely preserve circadian rhythmicity without disrupting the endogenous period, while inducing a significant phase advance in activity onset after fasting-induced torpor. Importantly, mice progressively resumed their normal activity levels in the days following torpor, even in the absence of external zeitgebers.

In designing these experiments, we considered three possible outcomes. The first scenario was that torpor would not adversely affect the circadian clock at all, with mice maintaining normally functioning rhythms and responses. The second possibility was that torpor could disrupt or desynchronize the clock; in this case, in the absence of external cues, mice would show irregular or fragmented activity patterns, reflecting a loss of circadian organization. The third scenario was that the reduced body temperature of torpor would slow down or freeze the transcription-translation feedback loop of the molecular clock. In this instance, the representation of time would slow down during torpor, causing a phase delay on arousal.

Our findings support the first scenario, as circadian rhythms remained largely intact, indicating that torpor does not lead to circadian desynchronization or disorganization. Rather than observing a phase delay as would be expected if the clock froze, we, in fact, identified a phase advance following fasting-induced torpor. Phase advance occurs when non-photic stimuli are presented in the inactive phase. In this instance, some aspect of torpor arousal appears to act as a phase advance stimulus. This indicates that the circadian system remains functionally responsive under hypometabolic conditions, retaining the ability to integrate physiological cues and adjust phase appropriately. The observed phase advance persisted across multiple days following torpor, suggesting that torpor-related events can exert a sustained, albeit modest, influence on endogenous clock mechanism, rather than produce a transient masking effect. While we cannot establish causality, the observed phase advance is likely driven by negative energy balance rather than by hypothermia per se, since under conditions of fasting, behavioral priorities shift toward food seeking, with arousal-promoting circuits actively suppressing sleep and redistributing activity across the circadian cycle in order to meet energetic demands.^21^ This interpretation is further supported by the lack of a statistically significant effect under synthetic torpor conditions, where hypothermia occurs in the absence of fasting.

We also observed a surge in wheel-running activity on the torpor day, occurring both immediately before torpor entry and following arousal. Similar increases in locomotor activity during food deprivation have been reported in mice, rats, and humans and are commonly interpreted as foraging-related behavior under conditions of negative energy balance^22^^,^^23^^,^^24^; therefore, the intense wheel running we observed before and after a torpor bout is most likely caused by food-seeking behavior. It is well documented that non-photic arousal stimuli, such as intense locomotory activity, applied during the second half of the subjective day can induce phase advance in both central and peripheral clocks.^13^^,^^14^^,^^25^ Therefore, our study suggests that emergence from torpor, or rather the behavioral changes it induces in the mouse, can act as a non-photic cue for the central circadian oscillator, as arousal and wheel-running occur during the late subjective day, when the clock is sensitive to such stimuli. A well-established pathway through which arousal-related signals can influence circadian timing involves serotonergic projections from the raphe nuclei to the suprachiasmatic nucleus (SCN).^26^^,^^27^ Serotonergic input to the SCN modulates circadian phase^28^^,^^29^ and is closely linked to behavioral activation and arousal. Another pathway classically involved in the non-photic entrainment of the SCN includes the activation of the intergeniculate leaflet (IGL) of the thalamus.^30^ The IGL collates non-photic information due to extensive innervation by arousal- and feeding-related systems, including serotonergic, noradrenergic, and orexinergic inputs.^31^^,^^32^^,^^33^^,^^34^^,^^35^ IGL neurons are additionally retinorecipient^36^ and thus send comprehensive non-photic information in the context of environmental illumination to entrain the SCN clock.^37^^,^^38^ Therefore, in the context of our study, hunger-driven locomotor activity during fasting-induced torpor may engage raphe-SCN signaling and/or activate the IGL-SCN geniculo-hypothalamic tract,^39^^,^^40^ providing a plausible mechanism through which fasting-associated arousal could contribute to the phase advance observed in the present study. It is worth noting that reported effects of fasting on circadian rhythms in rodents are variable across studies.^41^^,^^42^^,^^43^^,^^44^ Because core body temperature is often not measured, it is difficult to determine whether fasting-related circadian changes reflect metabolic state alone or are influenced by unrecognized torpor bouts, which are known to occur in fasted mice.^20^

In contrast, activity was significantly reduced on the day following torpor. Rather than indicating circadian disruption, this reduction likely reflects a transient redistribution of behavior, with mice spending more time feeding, drinking, and resting/sleeping to recover from the metabolic demands of arousal.

While cumulative food intake was stable over the long term (over a period of 6 days), daily consumption increased in the days following torpor, suggesting that although torpor provides an acute survival advantage during periods of food scarcity, it has a metabolic cost—particularly during arousal—requiring compensatory increases in feeding. Water intake patterns were similarly affected: intake was minimal during torpor, likely due to the inactivity of the mice, reduced insensible losses, and the concomitant reduction in food consumption. Notably, water intake was elevated in the days following torpor, which may reflect post-torpor recovery mechanisms. This rebound in water consumption is consistent with observations in other hibernators: marmots show high water intake upon emerging from hibernation^45^ and ground squirrels suppress thirst during torpor via osmolyte depletion and elevated vasopressin (AVP), resuming drinking upon arousal.^46^ More recently, Junkins and colleagues^47^ demonstrated that AVP and oxytocin pathways in the hypothalamic supraoptic nucleus are activated early during arousal in ground squirrels, promoting fluid retention and rehydration. Although mice do not experience the same depth of hypothermia, the post-torpor water intake we observed may reflect a similar rebound mechanism to restore fluid balance. Notably, this phenomenon has not previously been documented in mice and warrants further investigation.

Synthetic torpor produced effects broadly consistent with those observed during fasting-induced torpor in terms of circadian rhythmicity, with endogenous rhythms remaining robust and the free-running period unchanged. The absence of pre-torpor hyperactivity and post-torpor suppression of activity is consistent with the lack of fasting in this experiment and suggests that synthetic torpor may impose a lower physiological or metabolic burden than fasting-induced torpor. Although synthetic torpor did not produce a statistically significant phase shift, the direction of the effect was consistent with that observed during fasting-induced torpor, suggesting a shared influence on circadian timing that did not reach significance under the present conditions.

Overall, our findings indicate that mice maintain strong endogenous circadian rhythms while engaging in daily torpor. Both natural and synthetic torpor appear to leave the circadian clock largely intact, with fasting-induced torpor producing a significant phase advance in activity onset, despite the substantial reduction in core body temperature that occurred during a torpor bout, when mice reached ∼8°C–10°C below baseline. This resilience is consistent with recent evidence showing that the suprachiasmatic nucleus remains remarkably stable across a wide range of temperatures at the cellular level,^9^^,^^10^ suggesting that central circadian mechanisms can withstand pronounced hypothermia without loss of rhythmicity. Together, these findings provide *in vivo* evidence that circadian timekeeping remains functional during torpor in mice, even as other physiological processes are markedly slowed.

Behavioral and metabolic adjustments, such as advance in wheel-running activity, hunger-driven running, compensatory feeding, and rebound water intake, illustrate the complex integration of energy and fluid homeostasis with torpor. Together, these findings indicate that the circadian system in mice is remarkably resilient to the physiological perturbations associated with torpor, a feature that may facilitate the precise timing of arousal to coincide with the appropriate phase of the day-night cycle, suggesting that like torpor entry, emergence may also be under circadian regulation.

### Limitations of the study

This study has some limitations that should be considered. The sample size was modest, which may have limited the ability to detect smaller effects on circadian phase. Circadian rhythms were assessed through wheel-running activity, a robust and widely used measure, but one that reflects behavioral output rather than direct molecular clock function.

In fasting-induced torpor, hypothermia occurs alongside changes in energy balance and locomotor activity, both of which can influence circadian timing. Although the use of synthetic torpor helped to partially address this, it may not fully capture all aspects of natural torpor physiology. In addition, food removal and reintroduction may act as weak zeitgebers, although experimental design was used to minimize this influence.

Finally, the study focused on acute responses to single torpor episodes. Future work will be needed to determine whether repeated torpor bouts produce cumulative or longer-term effects on circadian regulation.

## Resource availability

### Lead contact

Further information and requests for resources should be directed to and will be fulfilled by the lead contact, Timna Hitrec (timna.hitrec2@unibo.it).

### Materials availability

This study did not generate new unique reagents.

### Data and code availability

• Data: data reported in this paper will be shared by the lead contact upon request.
• Code: this paper does not report original code.
• Additional information: any additional information required to reanalyze the data reported in this paper is available from the lead contact upon request.

## Acknowledgments

10.13039/100010269Wellcome Trust (WT) supported M.T.A. (308526/Z/23/Z), 10.13039/501100000265Medical Research Council (MRC) supported A.E.P. and M.T.A. (MR/W029138/1), and 10.13039/501100000268Biotechnology and Biological Sciences Research Council (BBSRC) supported M.E., T.H., A.E.P., and M.T.A. (BB/W007266/1).

## Author contributions

T.H., M.T.A., and A.E.P. designed the study. T.H., L.T., W.S.R.W., and M.E. performed the experiments. T.H. and L.T. analyzed the data. T.H. and L.T. prepared the figures. T.H., L.T., M.T.A., A.E.P., and L.C. wrote the initial draft of the manuscript. All authors assisted in editing the manuscript. We would like to thank Prof. Hugh D. Piggins for making available the TSE and Actimetrics acquisition systems and the ClockLab software to perform this study.

## Declaration of interests

The authors declare no competing interests.

## Declaration of generative AI and AI-assisted technologies in the writing process

During the preparation of this work the authors used ChatGPT edu (OpenAI) in order to help refine language and improve clarity. After using this tool/service, the author(s) reviewed and edited the content as needed and take full responsibility for the content of the published article.

## STAR★Methods

### Key resources table

**Table:** 

REAGENT or RESOURCE	SOURCE	IDENTIFIER
**Bacterial and virus strains**		

AAV2-hSyn-DIO-hM3Dq-mCherry	Krashes et al.^48^	Addgene, Cat.# 44361-AAV2

**Chemicals, peptides, and recombinant proteins**		

Clozapine N-oxide hydrochloride	Merck, Germany	Cat.# SML2304
4-hydroxytamoxifen	Tocris Bioscience, UK	Cat.# 0999
Ketaset (Ketamine HCl)	Zoetis	ATCvet code: QN01AX03
Domitor (medetomidine)	Zoetis	ATCvet code: QN05CM91
Antisedan (atipamezole)	Zoetis	ATCvet code: QV03AB90
Vetergesic	Ceva Animal Health	ATCvet code: QN02AE01

**Experimental models: Organisms/strains**		

C57BL/6 J mice	The Jackson Laboratory, USA	Strain #:000664
Fos2A-iCreERT2 mice	The Jackson Laboratory, USA	Strain #:030323

**Software and algorithms**		

Prism version 11	Graphpad, San Diego, CA	https://www.graphpad.com/scientific-software/prism/
Clocklab	Actimetrics	https://actimetrics.com/products/clocklab/

**Other**		

PhenoMaster acquisition system	TSE Systems, Bad Homburg, Germany	https://www.tse-systems.com/service/phenomaster/
Anipill temperature logger	BodyCAP	https://www.animals-monitoring.com/anipill-capsule-core-temperature-monitoring-system/

### Experimental model and study participant details

All experiments had the approval of the local University of Bristol Animal Welfare and Ethical Review Board and were conducted in accordance with the UK Animals (Scientific Procedures) Act (Project Licence: PP3581754).

A total of eighteen adult C57BL/6 J mice (aged 8–12 weeks, 11 females and 7 males) were used for the fasting-induced torpor study. No apparent sex-dependent differences were observed in the measured outcomes; therefore, data were not stratified by sex for statistical analyses. Animals were randomly assigned to experimental conditions according to the crossover experimental design. Seven homozygous females Fos2A-iCreERT2 adult mice, aged 8–12 weeks (TRAP2, Jax stock #030323)^49^^,^^50^ were used for the synthetic torpor experiments. Animals were housed individually in standard laboratory cages equipped with horizontal running wheels to monitor locomotor activity. The housing environment was maintained at a constant temperature of 22 ± 1°C with a 12-h light-dark (LD) cycle (lights on at zeitgeber time 0 [ZT0] and off at ZT12), except where specified otherwise. Food and water were provided *ad libitum* unless otherwise stated.

### Method details

#### Surgical procedures

All surgical procedures were performed under aseptic conditions in a dedicated surgical suite. Core temperature was maintained during anesthesia using a servo-controlled heat pad and a rectal temperature probe (Harvard Apparatus).

For core body temperature monitoring, a sterile AniPill temperature logger (TSE Systems) was implanted intraperitoneally (i.p) in all mice. Mice were anesthetized with isoflurane (4% induction, 1.5–2% maintenance in oxygen). A small midline incision was made in the abdominal wall, and the AniPill was carefully inserted into the peritoneal cavity. The muscle and skin layers were sutured with absorbable 5-0 Vicryl sutures, and mice were allowed to recover for at least one week before experimentation.

The mice for the synthetic torpor experiment (*n* = 7, TRAP2) underwent stereotaxic surgery to deliver an adeno-associated virus (AAV2-hSyn-DIO-hM3Dq-mCherry, a gift from Bryan Roth^48^ (www.addgene.org/44361). Injection pipettes were made from microcapillary glass (Sigma) on a vertical pipette puller (Harvard Apparatus). Pipettes were filled with mineral oil and then vector was back-filled.

Mice were anesthetized with ketamine (70 mg/kg i.p.) and medetomidine (0.5 mg/kg i.p.). Additional injections of anesthetic were administered as needed to maintain surgical depth of anesthesia. The planned incision site was shaved, and skin cleaned with iodine solution. Anesthetized mice were placed in a stereotaxic frame, the head was fixed in atraumatic ear bars, and skull position maintained by an incisor bar (David Kopf Instruments). The scalp was incised in the midline and burr holes made bilaterally at AP +0.4 mm, ML 0.5 mm with a drill (Neurostar Drill Robot). Bilateral injections of viral vector were made into the POA at depths of 5.1 and 5.0 mm relative to the surface of the brain using a microinjector (Nano-W wireless capillary microinjector, Neurostar). Each injection was 100 nL (titer 6 × 10^12^ vg/mL) delivered at a rate of 100 nL/min. The injection pipette remained in place for 1 min after the first injection and for 5 min after the second before removing.

Anesthesia was reversed with atipamezole (1 mg/kg, i.p.; Antisedan, Zoetis). For pain management, buprenorphine was given (0.1 mg/kg, s.c; Vetergesic, Ceva Animal Health). Postoperatively, mice were placed on a heating pad for recovery. They were housed individually during recovery and given softened food for the first 24 h post-surgery. Mice were allowed at least one week to recover from AniPill implantation and three weeks post-viral injection before further experimentation.

#### Torpor ‘TRAP’ procedure

TRAPing was conducted on mice that had received injection of the AAVs to express Cre-dependent excitatory DREADD (hM3Dq) transgenes into the preoptic area (POA). Mice were fasted from lights off and 12 to 14 h later, when in torpor, they received 4-hydroxytamoxifen (4-OHT, 50 mg/kg i.p) to genetically trap the active neurons^15^^,^^16^^,^^49^ in the POA to express hM3Dq. Food was returned 5 h later.

#### Drug preparation

The z-isomer of 4-OHT (Tocris Bioscience, Cat. No. 0999) was dissolved in Chen oil using the following method.^51^ First, 4-OHT was dissolved in neat ethanol at 20 mg/mL by shaking at 400 rpm and 37°C for 30–60 min until fully dissolved. Two parts Chen oil for every one-part ethanol was then added, and the ethanol was evaporated off using a vacuum centrifuge leaving a final solution of 10 mg/mL in Chen oil. Drug was prepared on the day of use, and if not used immediately, was kept in solution in the oil by shaking at 400 rpm at 37°C. Once in solution, the drug was protected from light.

Clozapine N-oxide hydrochloride (CNO, Merck, Cat. SML2304) was dissolved at 1 mg/mL in sterile water at room temperature. Aliquots were stored protected from light for up to 1 week.

#### Experimental design

The study comprised three separate experiments to investigate the effects of torpor on circadian rhythms and energy metabolism.

##### Experiment 1: Fasting-induced torpor

Mice (*n* = 6) were subjected to food deprivation starting at ZT12 to induce torpor (Figures 1A and 1B). Upon food deprivation, the standard LD cycle was discontinued, and mice were maintained in constant darkness (Torpor DD) to assess intrinsic circadian rhythm in absence of external photic cues. Food was reintroduced during constant darkness at Circadian Time 8 (CT8, as during constant darkness no Zeitgeber is present) on the day following the torpor bout. Using a cross-over design, the same mice were also assessed under constant darkness without food deprivation (Control DD). Wheel-running activity was recorded to monitor locomotor patterns.

##### Experiment 2: Fasting-induced torpor

Mice (*n* = 12) underwent a protocol similar to Experiment 1, with food removal at ZT12 (Figures 1A–1C). Mice were maintained in constant darkness either following arousal from torpor (Torpor DD) or after a matched period without manipulation (Control DD). A cross-over design ensured that all animals underwent both protocols. In this experiment, food was reintroduced during constant darkness, at CT12, after a 24-h fasting period. Food intake, water consumption, and locomotor activity were recorded (PhenoMaster, TSE Systems, Bad Homburg, Germany).

##### Experiment 3: Synthetic torpor

Following successful ‘TRAPing’ of POA neurons that were active during fasting induced torpor, synthetic torpor was induced by intraperitoneal injection of clozapine-N-oxide (CNO, 2 mg/kg) in fed mice (*n* = 7). CNO was administered at ZT18 to induce synthetic torpor (Figures 1A–1D). Following injection, mice were maintained in DD for the subsequent 7 days (Torpor DD). Also in this case, using a cross-over design, the same mice were also assessed under constant darkness without receiving CNO (DD Control). Wheel-running activity was recorded to monitor locomotor patterns.

### Quantification and statistical analysis

For Experiments 1 and 3, wheel-running activity was recorded (Actimetrics, Wilmette, IL, USA). Each wheel revolution was detected by a sensor and logged by an automated data acquisition system. Data were recorded in 30-s bins.

For Experiment 2, feeding, drinking, and locomotor activity were recorded (PhenoMaster). Food and water consumption were monitored to a precision of 0.01 g and 0.1 mL, respectively. Locomotor activity was recorded using the same approach as in Experiments 1 and 3, with each wheel revolution detected and logged. Data were recorded in 5-min bins.

Core body temperature was recorded at 1-min intervals using implanted sensors (AniPill). Torpor was defined as any period lasting ≥30 consecutive minutes with core temperature <33°C. A “torpor index” was calculated for each bout as the area under the curve (AUC) between the temperature trace and the 33°C threshold, integrating both duration and depth of the torpor bout.

Animals from Experiment 2 were weighed every other day, avoiding weighing on torpor day.

Statistical analysis was performed using GraphPad Prism v10.6.1. All datasets were tested for normality using the Shapiro-Wilk test.

To assess the overall effect of treatment (torpor induction by food deprivation or CNO injection), data from the 144 h (6 days) pre-treatment were compared to data from the 144 h (6 days) post-treatment using a two-tailed paired *t* test. This analysis was applied to cumulative wheel-running activity, food intake, and water intake. A *p*-value of <0.05 was considered statistically significant. In the figures (Figures 4A, 4B, 5A, 5B, and 6E), statistically significant comparisons are indicated as ∗∗∗∗ = *p* < 0.0001.

Day-to-day changes in activity (all experiments), food, and water intake (Experiment 2) were analyzed using a repeated measures one-way ANOVA (or mixed-effect model in case of missing datapoints, used for Experiment 1 and Experiment 3 activity data). Baseline values (Day −1) were derived from the average of the 6 days preceding treatment. Post-hoc comparisons were performed using Sidak’s test. For activity data, the following comparisons were made: baseline (Day −1) was compared with torpor day (Day 0) and with each of the subsequent five days (Days 1–5); additionally, torpor day (Day 0) was compared with each of the subsequent five days (Days 1–5). For food intake data, baseline (Day −1) was compared with each of the five days following torpor (Days 1–5) excluding day 0 as food was unavailable for that day as part of the experimental protocol for fasting-induced torpor. For water intake data, baseline (Day −1) was compared with torpor day (Day 0) and with each of the subsequent five days (Days 1–5). An adjusted *p* value of <0.05 was considered statistically significant. In the figures (Figures 4C, 4D, 5C, 5D, and 6F), statistically significant comparisons with Day −1 are indicated as ∗ = *p* < 0.05, ∗∗ = *p* < 0.01, ∗∗∗ = *p* < 0.001, ∗∗∗∗ = *p* < 0.0001, statistically significant comparison with Day 0 are indicated as ## = *p* < 0.01, ### = *p* < 0.001, #### = *p* < 0.0001.

ClockLab software (Actimetrics, v6) was employed for the extraction of circadian parameters (period determination and onset detection). For all experiments, the duration of the free-running period (τ) was calculated for both Torpor DD and Control DD conditions using the Lomb-Scargle periodogram within ClockLab, fitting the function over data collected from Day 2 to Day 5 post-manipulation. Values were compared between conditions using a paired *t* test. A *p*-value of <0.05 was considered statistically significant.

Activity onsets were automatically detected using ClockLab software. To quantify phase shifts, we calculated the deviation between the observed and the predicted activity onsets (“activity onset deviation”) over the 5 days following treatment. To obtain predicted values for activity onset, first, a baseline phase was defined as the mean activity onset time recorded during the 6 days preceding the onset of constant darkness (concurrent with food deprivation in experiments 1 and 2). For each post-manipulation day (Days 1–5), the predicted activity onset was extrapolated by projecting the baseline phase forward using the calculated τ (*Predicted Onset*_*n*_ = *Baseline* + *(n ∗τ))*. Finally, the activity onset deviation was calculated for each day by subtracting the actual onset detected by ClockLab from the predicted onset. Statistical differences in onset deviation were assessed using a two-way repeated measures ANOVA (or mixed-effect model in case of missing datapoints), with “Time” (5 levels corresponding to Days 1–5) and “Experimental Condition” (2 levels, corresponding to Torpor DD and Control DD) as factors. Sidak’s multiple comparisons test was then used to compare onset deviation between the two experimental conditions for each day. An adjusted *p*-value of <0.05 was considered statistically significant. In the figures (Figures 2D, 3D, and 6D), statistically significant post-hoc comparisons are indicated as ∗∗ = Sidak’s *p* < 0.01, ∗∗∗∗ = Sidak’s *p* < 0.0001.

To provide a quantitative evaluation of the magnitude of the experimental manipulation’s impact beyond statistical significance testing, effect size was calculated using Cohen’s f^52^ for both factors, as well as for their interaction. Cohen’s *f* and estimates of Partial-Eta Squared (η^2^_p_) values for factors “Time”, “Experimental Condition” and for their interaction for all three experiments are reported in Table S1.

For Experiment 2 animals, weight difference between Day −1 and Day 1, for both the Control DD and the Torpor DD conditions, were quantified as a percentage change from Day −1 to Day 1. Weight changes where then compared between the two conditions using a paired *t* test.

Data in the results section are presented as mean ± SEM. Details regarding the sample size for each statistical test are specified in figures’ legends.

## References

[1] Heldmaier G., Ortmann S., Elvert R. (2004). Respiratory Physiology and Neurobiology.

[2] Geiser F. (2004). Metabolic rate and body temperature reduction during hibernation and daily torpor. Annu. Rev. Physiol..

[3] Körtner G., Geiser F. (2000). The temporal organization of daily torpor and hibernation: circadian and circannual rhythms. Chronobiol. Int..

[4] Ruby N.F. (2003). Hibernation: when good clocks go cold. J. Biol. Rhythm..

[5] Ruf T., Geiser F. (2015). Daily torpor and hibernation in birds and mammals. Biol. Rev..

[6] Van Der Vinne V., Bingaman M.J., Weaver D.R., Swoap S.J. (2018). Clocks and meals keep mice from being cool. J. Exp. Biol..

[7] Colin Pittendrigh B.S., N Harvey C.E. (1954). ON TEMPERATURE INDEPENDENCE IN THE CLOCK SYSTEM CONTROLLING EMERGENCE TIME IN DROSOPHILA. Proc. Natl. Acad. Sci. USA.

[8] Narasimamurthy R., Virshup D.M. (2017). Molecular Mechanisms Regulating Temperature Compensation of the Circadian Clock. Front. Neurol..

[9] Enoki R., Kon N., Shimizu K., Kobayashi K., Hiro S., Chang C.P., Nakane T., Ishii H., Sakamoto J., Yamaguchi Y., Nemoto T. (2023). Cold-induced suspension and resetting of Ca2+ and transcriptional rhythms in the suprachiasmatic nucleus neurons. iScience.

[10] François P., Despierre N., Siggia E.D. (2012). Adaptive Temperature Compensation in Circadian Oscillations. PLoS Comput. Biol..

[11] Revel F.G., Herwig A., Garidou M.L., Dardente H., Menet J.S., Masson-Pévet M., Simonneaux V., Saboureau M., Pévet P. (2007). The circadian clock stops ticking during deep hibernation in the European hamster. Proc. Natl. Acad. Sci. USA.

[12] Williams C.T., Radonich M., Barnes B.M., Buck C.L. (2017). Seasonal loss and resumption of circadian rhythms in hibernating arctic ground squirrels. J. Comp. Physiol. B.

[13] Mrosovsky N. (1996). Locomotor activity and non-photic influences on circadian clocks. Biol. Rev. Camb. Phil. Soc..

[14] Mistlberger R.E., Antle M.C. (2011). Entrainment of circadian clocks in mammals by arousal and food. Essays Biochem..

[15] Ambler M., Hitrec T., Wilson A., Cerri M., Pickering A. (2022). Neurons in the Dorsomedial Hypothalamus Promote, Prolong, and Deepen Torpor in the Mouse. J. Neurosci..

[16] Hrvatin S., Sun S., Wilcox O.F., Yao H., Lavin-Peter A.J., Cicconet M., Assad E.G., Palmer M.E., Aronson S., Banks A.S. (2020). Neurons that regulate mouse torpor. Nature.

[17] Takahashi T.M., Sunagawa G.A., Soya S., Abe M., Sakurai K., Ishikawa K., Yanagisawa M., Hama H., Hasegawa E., Miyawaki A. (2020). A discrete neuronal circuit induces a hibernation-like state in rodents. Nature.

[18] Oelkrug R., Heldmaier G., Meyer C.W. (2011). Torpor patterns, arousal rates, and temporal organization of torpor entry in wildtype and UCP1-ablated mice. J. Comp. Physiol. B.

[19] Hitrec T., Luppi M., Bastianini S., Squarcio F., Berteotti C., Lo Martire V., Martelli D., Occhinegro A., Tupone D., Zoccoli G. (2019). Neural control of fasting-induced torpor in mice. Sci. Rep..

[20] Wheatley W.S.R., Marshall C.J., Taddei L., Hitrec T., Pickering A.E., Ambler M.T. (2025). The cold truth: torpor as a confound in studies of caloric restriction. J. Comp. Physiol. B.

[21] Northeast R.C., Vyazovskiy V.V., Bechtold D.A. (2020). Eat, sleep, repeat: the role of the circadian system in balancing sleep-wake control with metabolic need. Curr. Opin. Physiol..

[22] Hebebrand J., Exner C., Hebebrand K., Holtkamp C., Casper R.C., Remschmidt H., Herpertz-Dahlmann B., Klingenspor M. (2003). Hyperactivity in patients with anorexia nervosa and in semistarved rats: Evidence for a pivotal role of hypoleptinemia. Physiol. Behav..

[23] Exner C., Hebebrand J., Remschmidt H., Wewetzer C., Ziegler A., Herpertz S., Schweiger U., Blum W.F., Preibisch G., Heldmaier G., Klingenspor M. (2000). Leptin suppresses semi-starvation induced hyperactivity in rats: implications for anorexia nervosa. Mol. Psychiatr..

[24] Morse A.D., Russell J.C., Hunt T.W.M., Wood G.O., Epling W.F., Pierce W.D. (1995). Diurnal variation of intensive running in food-deprived rats. Can. J. Physiol. Pharmacol..

[25] Rosenwasser A.M., Dwyer S.M. (2001). Circadian phase shifting: Relationships between photic and nonphotic phase-response curves. Physiol. Behav..

[26] Glass J.D., Dinardo L.A., Ehlen J.C. (2000). Dorsal raphe nuclear stimulation of SCN serotonin release and circadian phase-resetting. Brain Res..

[27] Morin L.P. (1999). Serotonin and the regulation of mammalian circadian rhythmicity. Ann. Med..

[28] Mistlberger R.E., Antle M.C., Glass J.D., Miller J.D. (2000). Behavioral and Serotonergic Regulation of Circadian Rhythms. Biol. Rhythm Res..

[29] Cuesta M., Mendoza J., Clesse D., Pévet P., Challet E. (2008). Serotonergic activation potentiates light resetting of the main circadian clock and alters clock gene expression in a diurnal rodent. Exp. Neurol..

[30] Morin L.P. (2013). Neuroanatomy of the extended circadian rhythm system. Exp. Neurol..

[31] Webb I.C., Patton D.F., Hamson D.K., Mistlberger R.E. (2008). Neural correlates of arousal-induced circadian clock resetting: Hypocretin/orexin and the intergeniculate leaflet. Eur. J. Neurosci..

[32] Palus K., Chrobok L., Lewandowski M.H. (2015). Orexins/hypocretins modulate the activity of NPY-positive and -negative neurons in the rat intergeniculate leaflet via OX1 and OX2 receptors. Neuroscience.

[33] Chrobok L., Jeczmien-Lazur J.S., Pradel K., Klich J.D., Bubka M., Wojcik M., Kepczynski M., Lewandowski M.H. (2021). Circadian actions of orexins on the retinorecipient lateral geniculate complex in rat. J. Physiol..

[34] Smith V.M., Jeffers R.T., Antle M.C. (2015). Serotonergic enhancement of circadian responses to light: Role of the raphe and intergeniculate leaflet. Eur. J. Neurosci..

[35] Sanetra A.M., Palus-Chramiec K., Lewandowski M.H. (2021). Modulation of the Rat Intergeniculate Leaflet of the Thalamus Network by Norepinephrine. Neuroscience.

[36] Beier C., Zhang Z., Yurgel M., Hattar S. (2021). Projections of ipRGCs and conventional RGCs to retinorecipient brain nuclei. J. Comp. Neurol..

[37] Saderi N., Cazarez-Márquez F., Buijs F.N., Salgado-Delgado R.C., Guzman-Ruiz M.A., del Carmen Basualdo M., Escobar C., Buijs R.M. (2013). The NPY intergeniculate leaflet projections to the suprachiasmatic nucleus transmit metabolic conditions. Neuroscience.

[38] Fernandez D.C., Komal R., Langel J., Ma J., Duy P.Q., Penzo M.A., Zhao H., Hattar S. (2020). Retinal innervation tunes circuits that drive nonphotic entrainment to food. Nature.

[39] Challet E., Scarbrough K., Penev P.D., Turek F.W. (1998). Roles of Suprachiasmatic Nuclei and Intergeniculate Leaflets in Mediating the Phase-Shifting Effects of a Serotonergic Agonist and Their Photic Modulation during Subjective Day. J. Biol. Rhythm..

[40] Marchant E.G., Watson N.V., Mistlberger R.E. (1997). Both Neuropeptide Y and Serotonin Are Necessary for Entrainment of Circadian Rhythms in Mice by Daily Treadmill Running Schedules. J. Neurosci..

[41] Patton D.F., Mistlberger R.E. (2013). Circadian adaptations to meal timing: Neuroendocrine mechanisms. Front. Neurosci..

[42] Challet E., Mendoza J. (2010). Metabolic and reward feeding synchronises the rhythmic brain. Cell Tissue Res..

[43] Mendoza J. (2007). Circadian clocks: Setting time by food. J. Neuroendocrinol..

[44] Pickel L., Lee J.H., Maughan H., Shi I.Q., Verma N., Yeung C., Guttman D., Sung H.K. (2022). Circadian rhythms in metabolic organs and the microbiota during acute fasting in mice. Phys. Rep..

[45] Zatzman M.L., Thornhill G.V., Ray W.J., Ellersiek M.R. (1984). Seasonal changes of food and water consumption and urine production of the marmot, marmota flaviventris. Comp. Biochem. Physiol. A Physiol..

[46] Feng N.Y., Junkins M.S., Merriman D.K., Bagriantsev S.N., Gracheva E.O. (2019). Osmolyte Depletion and Thirst Suppression Allow Hibernators to Survive for Months without Water. Curr. Biol..

[47] Junkins M.S., Feng N.Y., Murphy L.A., Curtis G., Merriman D.K., Bagriantsev S.N., Gracheva E.O. (2024). Neural control of fluid homeostasis is engaged below 10°C in hibernation. Curr. Biol..

[48] Krashes M.J., Koda S., Ye C., Rogan S.C., Adams A.C., Cusher D.S., Maratos-Flier E., Roth B.L., Lowell B.B. (2011). Rapid, reversible activation of AgRP neurons drives feeding behavior in mice. J. Clin. Investig..

[49] Allen W.E., DeNardo L.A., Chen M.Z., Liu C.D., Loh K.M., Fenno L.E., Ramakrishnan C., Deisseroth K., Luo L. (2017). Thirst-associated preoptic neurons encode an aversive motivational drive. Science.

[50] DeNardo L.A., Liu C.D., Allen W.E., Adams E.L., Friedmann D., Fu L., Guenthner C.J., Tessier-Lavigne M., Luo L. (2019). Temporal evolution of cortical ensembles promoting remote memory retrieval. Nat. Neurosci..

[51] Guenthner C.J., Miyamichi K., Yang H.H., Heller H.C., Luo L. (2013). Permanent genetic access to transiently active neurons via TRAP: Targeted recombination in active populations. Neuron.

[52] Cohen J. (1988). Statistical Power Analysis for the Behavioral Sciences.

