# Supplementary material for: Clocks slide rather than freeze during torpor in the mouse
Source: iScience. 2026 Jun 17;29(7):116402. doi: 10.1016/j.isci.2026.116402 (PMC13310930; doi:10.1016/j.isci.2026.116402)
Supplement: Document S1. Figures S1–S3 and Table S1 [file mmc1.pdf]

**iScience, Volume *29***

## Supplemental information

# Clocks slide rather than freeze during torpor in the mouse

**Timna Hitrec, Ludovico Taddei, Lukasz Chrobok, Megan Elley, William S.R. Wheatley, Anthony E. Pickering, and Michael T. Ambler**

## Supplementary Figures and Tables

| Experiment | Statistical test | Factor | $\eta^2_p$ | Cohen's *f* |
|---|---|---|---|---|
| **I** | Mixed Effect Model | Experimental condition | 0.19 | 0.48 |
| | | Time | 0.11 | 0.36 |
| | | Interaction | 0.22 | 0.52 |
| **II** | ANOVA | Experimental condition | 0.71 | 1.57 |
| | | Time | 0.56 | 0.56 |
| | | Interaction | 0.24 | 0.24 |
| **III** | Mixed Effect Model | Experimental condition | 0.27 | 0.60 |
| | | Time | 0.10 | 0.33 |
| | | Interaction | 0.17 | 0.46 |

**Table S1 – Comparison of standardised effect sizes (Cohen's *f*) across experiments**
To ensure comparability between the Mixed-Effect Models (Experiments I and III) and the repeated measures ANOVA (Experiment II), effect sizes are reported as Cohen's *f*. values were derived from partial eta-squared ($\eta^2_p$) estimates calculated via F-statistics. Effect magnitudes are interpreted according to Cohen's (1988) benchmarks: small (0.10), medium (0.25), and large (0.40).

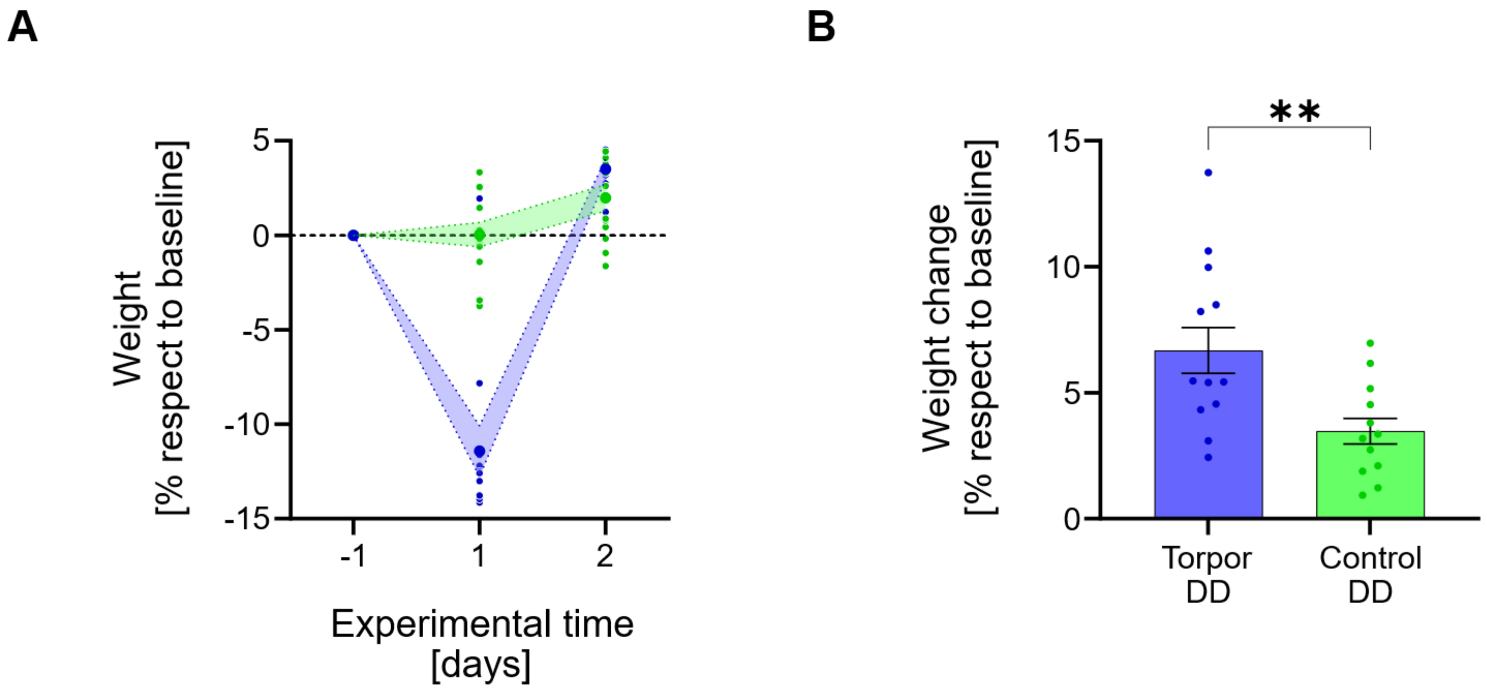


**Figure S1 – Experiment 2 weight progression**

**Panel A**: weight progression of animals during Experiment 2. On the X-axis, experimental day, with respect to the time of treatment are indicated. Blue symbols represent Torpor DD; green symbols represent Control DD. Data are represented as mean ± SEM. **Panel B**: post-treatment weight comparison between animals in Torpor DD and the same animals in Control DD. Data are shown as mean ± S.E.M., dots represent individual values. Data were compared between the two groups through a paired t-test. Torpor DD: 6.81±0.98% vs. Control DD: 3.51±0.55%; paired t-test: $t_{(11)}=4.021$, $p=0.002$. ** = $p < 0.01$.

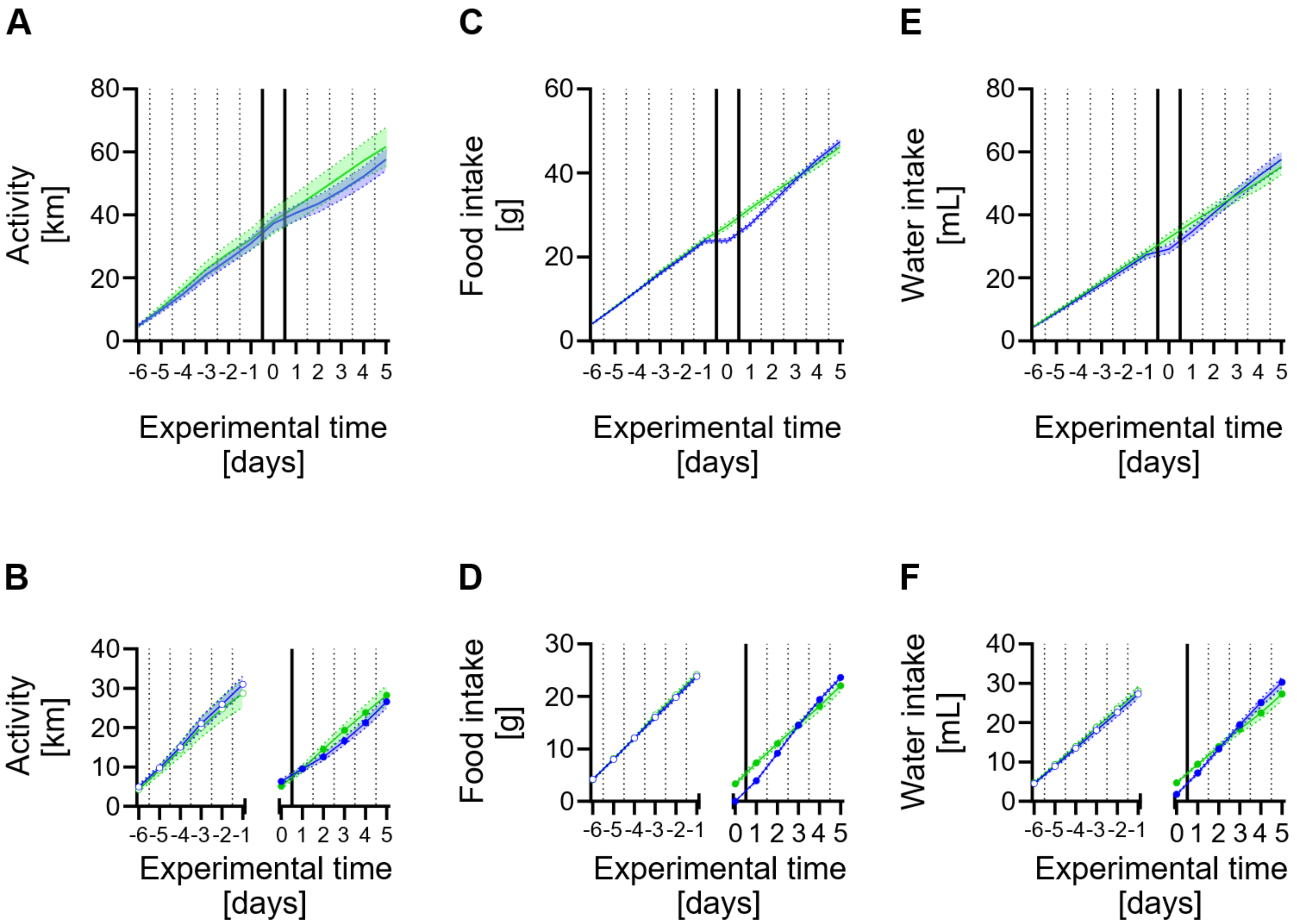


**Figure S2 – Comparison of cumulative data progression over time in Experiment 2 animals.** **Panel A**: cumulative data regarding locomotor activity in animals from Experiment 2. Data are presented as mean values for 24-hours bins (starting from ZT12, the time at which treatment was administered). Blue line represents food deprivation in constant darkness (Torpor DD), green line represents normal feeding in constant darkness (Control DD). The coloured areas represent the standard error of the mean. The black vertical solid lines indicate the bin corresponding to treatment day; vertical dotted lines represent each day CT12. The six days preceding and the six days following the time of food deprivation (or of sham treatment) are represented. **Panel B**: same data presented as detailed comparison between the six days preceding and the six days following the time of food deprivation (or of sham treatment). Blue dots represent Torpor DD datapoints, green dots represent Control DD datapoints. Empty dots represent the first 6 days, filled dots represent the last 6 days. The coloured areas represent the standard error of the mean. The black vertical solid line indicates the end of the bin corresponding to treatment day; vertical dotted lines represent each day CT12. **Panel C&D**: equivalent plots of cumulative food intake in the six days preceding and following the time of food deprivation in Experiment 2. **Panel E&F**: equivalent plots of cumulative water intake.

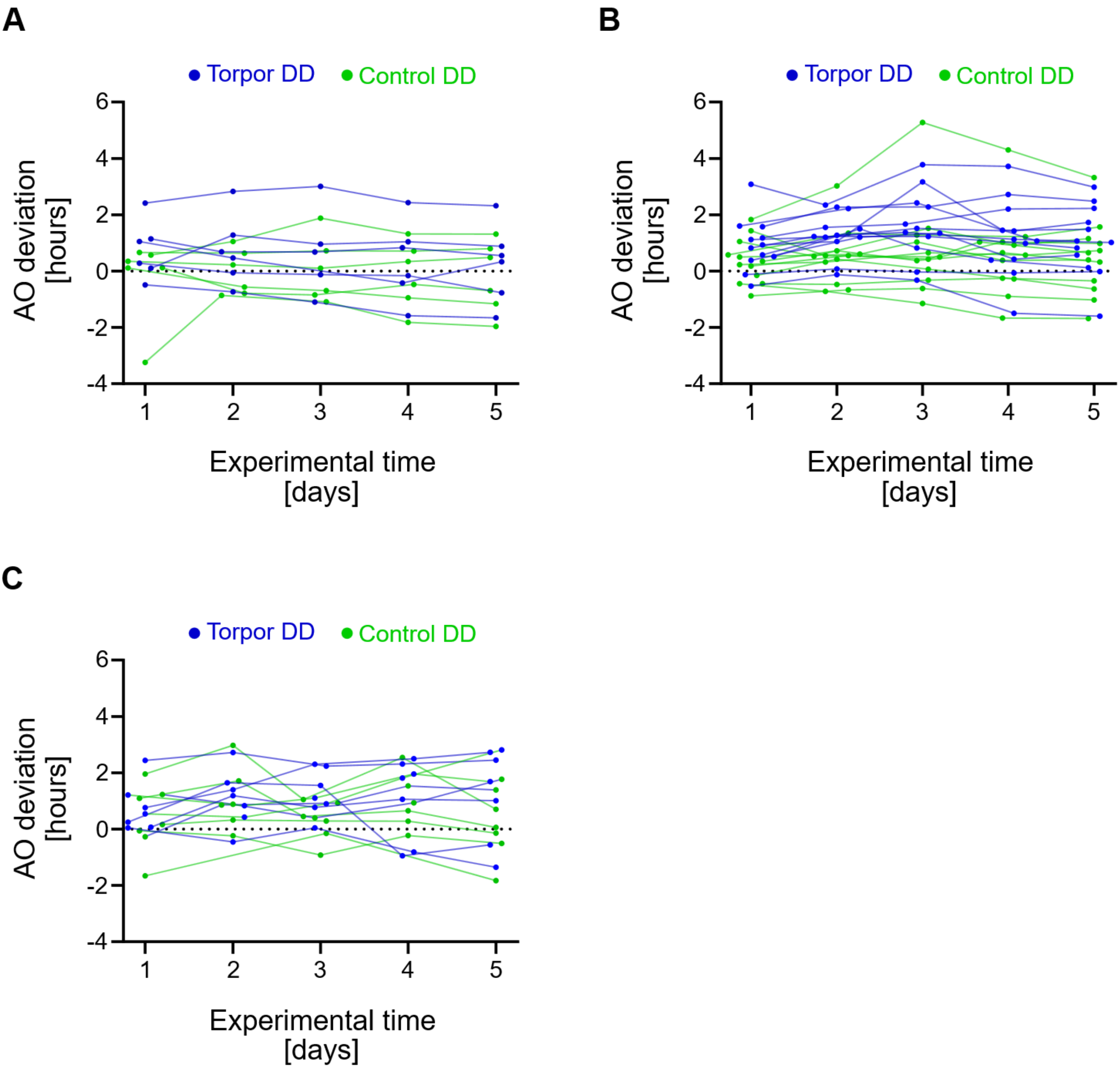


**Figure S3 – Individual datapoints for activity onset deviation analysis.**
**Panel A**: activity onset deviation from predicted values in the first 5 days of constant darkness in the Torpor DD condition and in the Control DD condition in Experiment 1 (n = 6). Blue and green dots represent individual values. Detail of statistical analysis and results can be found in Figure 2D. **Panel B**: equivalent plot for Experiment 2 (n = 12). Detail of statistical analysis and results can be found in Figure 3D. **Panel C**: equivalent plot for Experiment 3 (n = 7). Detail of statistical analysis and results can be found in Figure 6D.
